# Enhanced gallbladder cancer detection via active and self-supervised learning integration: Innovating B-ultrasound image analysis

**DOI:** 10.1371/journal.pone.0330781

**Published:** 2025-09-16

**Authors:** Jia Li, Yu-Qian Zhou

**Affiliations:** College of Applied Mathematics, Chengdu University of Information Technology, Chengdu, Sichuan, China; Sichuan University, CHINA

## Abstract

Gallbladder cancer, a common yet often under diagnosed malignancy, is typically characterized by late detection and a poor prognosis. The rise of deep learning has introduced new methods for its early identification through B-ultrasound imaging, but there are still challenges of inefficient data labeling and feature extraction. This paper introduces a novel classification algorithm, ASGBC, intended to tackle related challenges in diagnosing gallbladder cancer using B-ultrasound images. Firstly, we combine active learning with self-supervised learning to decrease the reliance on labeled data. Secondly, we introduce the MsHop module, which effectively captures the fine textures and patterns in ultrasound images through the integration of multi-scale and high-order information, thereby improving diagnostic accuracy. Additionally, we develop a dual-branch loss function that leverages data correlation and clustering features to enhance feature extraction and model stability. The experiments on a gallbladder ultrasound dataset have confirmed the effectiveness of our algorithm, achieving an accuracy of 0.884, a specificity of 0.932, and a sensitivity of 0.912—outperforming existing methods. The results exhibit lower variance, indicating improved model stability. Furthermore, the findings demonstrate that using active learning, one can achieve comparable results to those from the full dataset with only 35% of the data, reducing annotation costs and increasing model learning efficiency. Further research will concentrate on refining the algorithm for wider clinical use and identifying additional features that may further improve diagnostic accuracy.

## Introduction

Gallbladder cancer (GBC) is a highly malignant tumor with a very poor prognosis, posing a significant threat to global public health. Although bile duct malignancies are relatively rare, GBC is the most common and aggressive among them, particularly affecting women [1]. Ranked 22nd among all common cancers, the incidence rate of GBC is higher in women (20th) compared to men (23rd) [2]. It is the sixth leading cause of cancer-related deaths among various malignancies. According to the GLOBOCAN 2020 report, in 2020, there were 115,949 new cases and 84,695 deaths worldwide, encompassing all ages and genders. Asia has the highest incidence and mortality rates, accounting for 71.9% and 75.0% of the global figures, respectively [3]. Therefore, early detection is crucial for effective treatment and improving patient survival rates.

Given the anatomical location of GBC and its asymptomatic nature or symptoms that mimic other diseases, early detection is challenging. It is often discovered incidentally after gallbladder removal surgery for other indications [4]. A study conducted in 2021 showed that less than 15% of GBC cases diagnosed in the United States between 2013 and 2017 were at a localized stage. The majority were diagnosed at later stages, with 38.7% at the regional stage and 44.4% at the distant stage [5].

Ultrasound is the primary diagnostic tool for gallbladder diseases due to its safety, cost-effectiveness, and ease of use. It is commonly used for the initial assessment of suspected gallbladder diseases. In resource-limited countries, it is often the only imaging examination available to patients with abdominal diseases. However, ultrasound images can be compromised by noise artifacts, such as speckle noise, which degrades image quality—a problem not commonly found in CT, MRI, PET, and SPECT imaging modalities. Detecting malignant gallbladders is more challenging due to the lack of clear boundaries or morphological features compared to normal and benign gallbladder areas. Therefore, the accuracy of ultrasound diagnosis largely depends on the experience of the sonographer and the diagnosing physician [6]. While certain characteristics aid in identifying GBC on ultrasound images, differentiating it from other abnormalities in the early stages remains difficult [7]. Thus, analyzing ultrasound images to establish and understand the characteristics of malignant gallbladder tumors is essential for enhancing the recognition and differentiation of GBC, ultimately preventing under-treatment and over-treatment.

The evolution of deep learning has opened new avenues for medical diagnostics using ultrasound images. A plethora of researchers has applied deep learning techniques to analyze ultrasound images across different organs, thereby assisting in medical diagnosis. This encompasses a range of conditions including breast tumors [8,9], prostate nodules [10,11], thyroid nodules [12,13], ocular diseases [14,15], pulmonary conditions [16,17], and fetal imaging [18,19]. However, compared with CT and MRI, the studies based on B-ultrasound images is much less, and there is even less about GBC.

Self-supervised learning(SSL) and active learning(AL) hold significant potential in medical image analysis, enhancing the model’s learning capability and diagnostic accuracy through intelligent data selection and utilization of the data’s inherent structure [20,21]. SSL trains models by predicting transformations or attributes of the data without external labeling, making full use of unlabeled image data [22–24]. AL allows models to identify and request labeling for the most uncertain samples, optimizing the learning process with limited expert resources [25,26]. These methods can strengthen the model’s ability to recognize subtle features in medical images and improve generalization to different lesion types, which is crucial for increasing diagnostic efficiency and accuracy. But the majority of existing studies have relied on supervised learning methodologies, necessitating the use of labeled datasets.

At present, there is no research on using AL in B-ultrasound images. A smaller subset of research has delved into SSL algorithms to pre-train models for extracting features from B-mode ultrasound images. Researchers Nguyen et al. [27] assessed the efficacy of the BYOL algorithm [28] for classifying breast ultrasound images using a public dataset of breast expert data. Mishra et al. [29] pre-trained an encoder-decoder architecture to perform deterministic edge detection or segmentation tasks without the need for machine learning. Experiments conducted on two public datasets demonstrated that SSL enhances performance, particularly when there is a scarcity of labeled training data. Zhao and Yang [30] utilized the public TN-SCUI2020 dataset to preprocess classifiers for distinguishing between benign and malignant thyroid nodules. Researchers Jiao et al. [31] and Chen et al. [32] applied Self-Supervised Learning to obstetric-related ultrasound image analysis tasks. Liu et al. [33] pre-trained an encoder-decoder model for downstream tasks of classifying gastrointestinal stromal tumors from endoscopic ultrasound images.

It can be found that using deep learning models to analyze ultrasound images poses a major challenge. Firstly, unlike MRI or CT, ultrasound images tend to have lower image quality and are susceptible to noise and sensor artifacts, existing feature extractors, which are primarily designed for natural images, are more likely to learn from false textures and fail to truly capture the characteristics of GBC. Moreover, most existing classification algorithms for GBC rely on fully supervised learning, requiring all data to be labeled. Algorithms proposed by Basu et al. [34,35] are based on unsupervised and self-supervised learning, but they all necessitate the use of B-ultrasound video information, leading to high training resource demands. The integration of AL and SSL offers a promising yet unexplored path. To reduce training costs while enhancing the accuracy of diagnosing GBC with B-ultrasound images, this paper introduces a classification algorithm for GBC that combines AL and SSL, called ASGBC. The specific contributions are as follows:

1. Integration of AL and SSL: We implement AL prior to SSL to preselect data with high information value. This proactive selection reduces the training time for the classification model, decreases the demand for computational resources, and lowers deployment costs.
2. Design of the MsHop Module: By extracting multi-scale and high-order information from images simultaneously, it comprehensively encodes tumor characteristics, ensuring a detailed and accurate feature representation.
3. Design of a Dual-Branch Loss Function: It considers both data correlation and clustering features, making feature extraction more refined and robust, thereby improving the model’s predictive accuracy and stability.

## Related works

**Deep learning applications for gallbladder cancer diagnosis.** With the development of deep learning technology, its application in medical image analysis has provided new perspectives for improving the accuracy and efficiency of diagnosing GBC. Recent studies have introduced several innovative methods in this domain. Lian et al. [36] presented an automatic segmentation method for gallbladder and gallstone regions in ultrasound images, integrating an improved Otsu algorithm, anisotropic diffusion, global morphology filtering, a parameter-adaptive pulse-coupled neural network (PA-PCNN), and locally weighted regression smoothing (LOESS) for enhanced accuracy and efficiency. Jeong et al. [37] developed a deep learning-based decision support system (DL-DSS) that significantly improved the performance of gallbladder polyp diagnosis on ultrasound through transfer learning, demonstrating that the diagnostic performance assisted by DL-DSS was superior to that of individual radiologists. Kim et al. [38] enhanced the classification accuracy of gallbladder polyps less than 20 millimeters by using an ensemble convolutional neural network model, showing the potential of deep learning to improve clinical diagnostic specificity. Basu et al. [39] developed GBCNet, a CNN-based model that excels in GBC detection from ultrasound images. It overcomes challenges of low image quality and spurious textures through a novel ROI extraction method, a multi-scale second-order pooling architecture, and a curriculum inspired by human visual acuity, outperforming both state-of-the-art models and expert radiologists. Basu et al. [34] also introduced an innovative unsupervised contrastive learning (UCL) framework that uses hard negatives from temporally distant frames within the same ultrasound video, along with a hardness-sensitive negative mining curriculum, to enhance image representation learning for gallbladder malignancy detection, achieving higher accuracy than state-of-the-art techniques. Shuvo and Chowdhury [40] proposed a method for GBC classification using an ensemble of well-known convolutional neural network models, significantly enhancing classification accuracy.

**Active learning.** Existing AL approaches can be divided into two main groups: distribution-based and uncertainty-based methods. AL has emerged as a pivotal paradigm for enhancing the efficiency of model training by selectively labeling informative data points. Sener and Savarese [41] redefined AL as core-set selection, focusing on choosing a diverse subset of data. Pinsler et al. [42] offered a Bayesian batch AL method that approximates the posterior for model parameters, enabling scalable AL. Sinha et al. [43] introduced Variational Adversarial Active Learning (VAAL), leveraging a VAE and adversarial network for representation learning. Xie et al. [44] proposed Energy-based Active Domain Adaptation (EADA), utilizing energy-based models to reduce domain gaps. Cabannes et al. [45] presented Positive Active Learning (PAL), a framework that integrates SSL with AL by querying semantic relationships. These works collectively advance the field by addressing challenges in labeling efficiency, representation learning, and scalability in various learning scenarios.

**Self-supervised learning.** SSL has made significant strides in recent years, with various innovative approaches proposed to learn meaningful representations without labeled data. The introduction of frameworks like SimCLR [46], MoCo [47], and BYOL [28] has revolutionized the field by simplifying the learning process and enhancing the quality of learned representations. These methods focus on maximizing the similarity between augmented versions of the same image, effectively learning to discriminate between different instances. Additionally, SwAV [48] introduced an online clustering approach that contrasts cluster assignments, further improving the efficiency and scalability of SSL. Barlow Twins [49] presented a redundancy reduction principle to ensure informative yet invariant representations. Innovative works by He et al. [50], Chen et al. [51], and Bao et al. [52] significantly advanced the state-of-the-art by introducing masked autoencoders (MAE) and their scalable variants, demonstrating the efficacy of reconstructing masked image patches for learning meaningful representations. The seminal paper by Huang et al. [53] on Vision Transformers has further catalyzed research in this domain, showing that self-supervised methods can be effectively scaled up with the right architectural choices. The recent breakthrough by Mishra et al. [54] presented a simple yet efficient contrastive masked autoencoder, highlighting the complementary nature of contrastive learning and MAE. Furthermore, the work of Oquab et al. [55] on DINOv2 underscores the potential of SSL to produce versatile visual features that are competitive with weakly-supervised models across diverse tasks. Collectively, these works have pushed the boundaries of unsupervised visual representation learning, achieving competitive results with supervised counterparts and opening new avenues for research in computer vision.

In summarizing the related work on diagnosing GBC on B-ultrasound images using deep learning, the majority of explorations have been based on deep learning schemes that rely solely on labeled data. While some methods have indeed harnessed the information stored in unlabeled data through SSL [34,35], they all necessitate the use of video-level data rather than individual B-ultrasound images. Furthermore, no methods have addressed the reduction of labeling costs and computational resources through the use of AL. In this paper, we combine AL with SSL, effectively saving on training-related costs and enhancing the model’s accuracy and robustness by improving the feature extraction network and loss function within SSL.

## Method

### Overview of the framework

**Fig 1:**
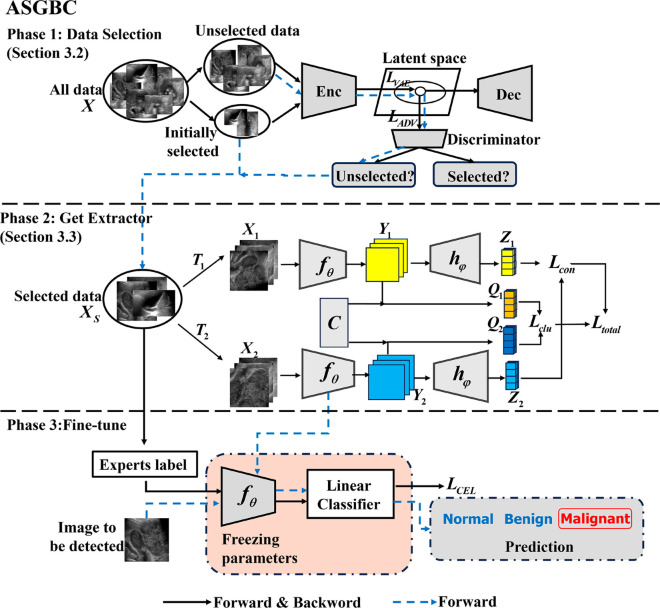
Framework of the proposed algorithm. (The proposed method, ASGBC, integrates AL in the first phase and SSL in the second phase. This integration helps to reduce the required training resources and labeling effort to less than 35% of the dataset.)

Our proposed approach ASGBC combines AL and SSL to minimize training expenses and the labor of labeling, while enhancing the model’s precision and stability through the integration of multi-scale, high-order information, as well as features that account for image correlation and clustering. The flowchart of our algorithm is illustrated as shown in Fig 1.

For details on the specific algorithmic process, please refer to Algorithm 1. Initially, AL is employed to train the data selection network, which selects the most informative samples *X*_*S*_ from the pool of unlabeled data *X*(Details can be found in section Following this, a feature extractor$f_{\theta }$ that incorporates multi-scale modules and high-order pooling modules is trained using the selected data *X*_*S*_ in conjunction with SSL techniques(For more information, please refer to section Get extractor). The features learned can then be applied to fine-tune various downstream tasks, including but not limited to classification, localization, and detection. For this paper, the downstream task focuses on the classification of GBC in B-ultrasound images. A classifier with a single linear layer is appended to the feature extraction backbone, with the feature extractor’s parameters frozen to retain the generalized features learned. The classifier is subsequently fine-tuned using a modest amount of labeled B-ultrasound data to achieve the final classification model.

**Algorithm 1. ASGBC.**

1: **Input:** Unlabeled dataset *X*

2: **Output:** Trained GBC classification model

3: **Phase 1: Data Selection (Active Learning based on VAAL)**

4: Randomly select 5% data from *X* to initialize as *X*_*S*_, and $X_{U}=X-X_{S}$.

5: **for** epoch =1 to epochs **do**

6:   Sample $x_{S}\sim X_{S}$, Sample $x_{u}\sim X_{u}$

7:   Compute $L_{\text{VAE}}^{trd}$ and $L_{\text{VAE}}^{adv}$ by using Eqs (1) and (2) respectively

      (*Eq (1)**: VAE reconstruction loss;* *Eq (2)**: adversarial loss for discriminator*)

8:    Compute $L_{\text{VAE}}$ by using Eq (4)

      (*Eq (4)**: Combined VAE loss integrating reconstruction and adversarial components*)

9:    Update VAE by descending stochastic gradients: $\theta_{\text{VAE}}^{\prime }=\theta_{\text{VAE}}-\alpha_{1}\nabla L_{\text{VAE}}$

10:    Compute *L*_*D*_ by using Eq (3)

      (*Eq (3)**: Discriminator loss for active learning*)

11:    Update *D* by descending its stochastic gradient: $\theta_{D}^{\prime }=\theta_{D}-\alpha_{2}\nabla L_{D}$

12:    Train and update *T*: $\theta_{T}^{\prime }=\theta_{T}-\alpha_{3}\nabla L_{T}$

13: **end for**

14: Select samples (*X*_*s*_) with $min_{p-5}\{\theta_{D}(z_{U})\}$

      (*Select samples with highest uncertainty from unlabeled pool*)

15: $X_{S}\leftarrow X_{S}⋃X_{s}$

16: $X_{U}\leftarrow X_{U}-X_{s}$

17: **Phase 2: Feature Extractor Training (Self-Supervised** **Learning)**

18: **for** epoch =1 to epochs **do**

19:   **for** batch =1 to batchs **do**

20:    Sample a batch data $x_{s}\sim X_{S}$

21:    Calculate two different enhancements $X_{1}=T_{1}(x_{s})$ and $X_{2}=T_{2}(x_{s})$ respectively

22:    Extract the features $Y_{1}=f_{\theta }(X_{1})$ and $Y_{2}=f_{\theta }(X_{2})$ using feature extractor $f_{\theta }$

23:    Compute embeddings $Z_{1}=h_{\varphi }(Y_{1})$ and $Z_{2}=h_{\varphi }(Y_{2})$ using expander $h_{\varphi }$

24:    Compute encodings *Q*_1_ and *Q*_2_ using Eq (17)

25:    Compute $L_{con}(Z_{1},Z_{2})$ using Eq (11)

      (*Eq (11)**: Contrastive loss for feature invariance*)

26:    Compute $L_{clu}(Y_{1},Y_{2})$ using Eq (15)

      (*Eq (15)**: Clustering loss for feature discriminability*)

27:    Compute *L*_*total*_ using Eq (5)

      (*Eq (5)**: Combined loss integrating contrastive and clustering objectives*)

28:    The loss function *L*_*total*_ is minimized to train parameters *θ* and φ

29:   **end for**

30: **end for**

31: **Phase 3: Supervised Fine-tuning**

32: Annotate the selected data *X*_*S*_ for classification

      (*Perform manual annotation on the selected samples*)

33: Freeze the weights of $f_{\theta }$

      (*Fix the pre-trained feature extractor parameters*)

34: Fine-tune the linear classification layer with supervised training on *X*_*S*_ to obtain the GBC classification model

      (*Only update the final classification layer with labeled data*)

35: **return** Trained GBC classification model

### Data selection

This section explains how we utilize AL to select the most informative data. Although our method is compatible with any AL technique, we draw inspiration from the Variational Adversarial Autoencoder (VAAL) [43]. We employ a *β*-Variational Autoencoder (*β*-VAE) [56] and an adversarial network [57] to implicitly learn the sampling mechanism. The *β*-VAE is responsible for learning the latent space representation of the data, while the adversarial network discerns between labeled and unlabeled data. A minimax game is played between the *β*-VAE and the adversarial network, where the *β*-VAE aims to deceive the adversarial network into believing all data points originate from the labeled data pool. Concurrently, the adversarial network strives to differentiate between them within the latent space. For specific algorithm steps, refer to Algorithm 1 (Phase 1). This phase does not require any labels; instead, it is entirely based on the intrinsic characteristics of the data.

Among them, the transduction of VAE represents a transformational representation learning objective function is:

$$\begin{array}{ccc} L_{\text{VAE}}^{trd} & = & 𝔼[\log p_{\theta }(x_{S}|z_{S})]-\beta D_{\text{KL}}(q_{\phi }(z_{S}|x_{S})||P(z))\\ & & +𝔼[\log p_{\theta }(x_{U}|z_{U})]-\beta D_{\text{KL}}(q_{\phi }(z_{U}|x_{U})||P(z)), \end{array}$$ (1)

VAE’s antagonism represents the learning objective function is:

$$L_{\text{VAE}}^{adv}=-𝔼[\log(D(q_{\phi }(z_{S}|x_{S})))]-𝔼[\log(D(q_{\phi }(z_{U}|x_{U})))],$$ (2)

training objective function of countermeasure network is:

$$L_{D}=-𝔼[\log(D(q_{\phi }(z_{S}|x_{S})))]-𝔼[\log(1-D(q_{\phi }(z_{U}|x_{U})))],$$ (3)

complete VAE objective function of VAAL is:

$$L_{\text{VAE}}=\lambda_{1}L_{\text{ VAE}}^{trd}+\lambda_{2}L_{\text{VAE}}^{adv},$$ (4)

where 𝔼 is mathematical expectation; $q_{\phi }$ and $p_{\theta }$ are encoders and decoders, respectively, and are parameterized by parameters *ϕ* and *θ*; *P*(*z*) is the selected prior distribution, usually the unit Gaussian distribution; β is the Lagrange multiplier of the optimization problem; $D_{\text{KL}}$ indicates Kullback-Leibler divergence; *D* is the differentiator of the adversarival network; $\lambda_{1}$ and $\lambda_{2}$ are superparameters, which are used to determine the effect of each component in learning effective variational antagonism representation.

### Get extractor

In this section, we will describe how to employ an enhanced SSL algorithm to train a feature extractor $f_{\theta }$ for gallbladder B-ultrasound images. The framework of this SSL algorithm is illustrated in Fig 1 (Phase 2). Similar to traditional contrastive learning methods, it features two branches for data augmentation, with distinctions in the following two aspects.

1. The feature extractor $f_{\theta }$ is specially designed, incorporating a multi-scale high-order feature extraction module(MsHop) into the Resnet backbone. This allows for the integration of multi-scale and high-order information from images, leading to more accurate extraction of GBC features.

2. A unique dual-branch loss function is designed, integrating the correlation and clustering features of the feature maps. It optimizes the model from multiple perspectives, enhancing the feature extraction capability while also strengthening the model’s stability.

Algorithm 1 (Phase 2) provides a detailed step-by-step guide for training a feature extractor using an improved self-supervised algorithm. Given a batch of images *x*_*S*_, two different batches of views *X*_1_ and *X*_2_ are generated by transformations *T*_1_ and *T*_2_, respectively, and then encoded into representations *Y*_1_ and *Y*_2_ using a feature extractor $f_{\theta }$. These representations are processed by two different branches, which handle feature encoding differently, resulting in two losses *L*_*con*_ and *L*_*clu*_ through intra-batch denormalization and exchange prediction, respectively. The final loss is a combination of these two losses:

$$L_{total}(Y_{1},Y_{2},Z_{1},Z_{2})=L_{con}(Z_{1},Z_{2})+\alpha L_{clu}(Y_{1},Y_{2}),$$ (5)

where *α* is a hyperparameter.

We will now proceed to detail the MsHop module and the dual-branch loss function.

**Fig 2:**
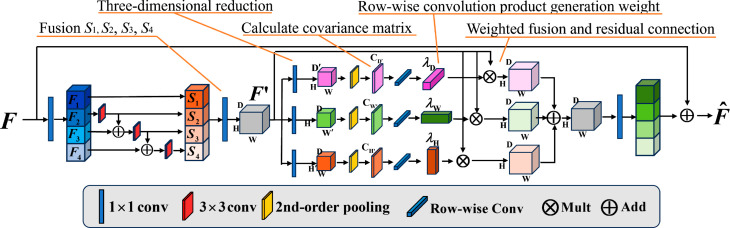
Framework of MsHop.

The core concept of multi-scale design is to establish hierarchical residual connections within a single residual block, representing multi-scale features and increasing the receptive field of each network layer. Higher-order pooling aims to use all three feature dimensions to learn a robust second-order covariance representation, thereby enhancing the accuracy of diagnosing GBC in ultrasound images. The MsHop module integrates these two approaches, as shown in Fig 2. This module takes into account both multi-scale information and the height, width, and channel dimensions to enhance the learned second-order statistical information.

Firstly, we introduce the MsHop module. Its computational process is depicted in Fig 2

Assuming the input feature map *F* has a size of W×H×D, it is divided into four subsets $F_{1},F_{2},F_{3},F_{4}$ along the depth direction. Each subset *F*_*i*_ has a size of W×H×D/4 and is processed by a 3x3 convolution *K*_*i*_, resulting in output *S*_*i*_. Except for *F*_1_, each *F*_*i*_ is added to the output *S*_*i*−1_ of the previous filter bank and then input to the next filter bank ${\text{Conv}}_{3\;\times \;3}^{i}$. This process can be expressed as:

$$S_{i}=\left\{ \begin{array}{cc} {\text{Conv}}_{3\;\times \;3}^{i}(F_{i}), & \text{if }i=1,\\ {\text{Conv}}_{3\;\times \;3}^{i}(F_{i}⊕S_{i-1}), & \text{if }i=2,3,4, \end{array}\right.$$ (6)

where ${\text{Conv}}_{3\;\times \;3}^{i}$ represents the *i*-th group of 3×3 convolutions. Finally, all *S*_*i*_ are merged (e.g., by concatenation) and fused by a 1×1 convolution ${\text{Con}}_{1\;\times \;1}$, yielding the middle feature map $F^{\prime }$:

$$F^{\prime }={\text{Con}}_{1\;\times \;1}([S_{1},S_{2},S_{3},S_{4}]).$$ (7)

Assume the feature map $F^{\prime }$ has size of W×H×D, reduce the feature dimension to $W\;\times \;H\;\times \;D^{\prime }$, $W^{\prime }\;\times \;H\;\times \;D$, and $W\;\times \;H^{\prime }\;\times \;D$ by three 1×1 convolution layers respectively,

$$F_{W^{\prime }}^{\prime }={\text{Con}}_{1\;\times \;1}^{W}(F^{\prime }),\;F_{D^{\prime }}^{\prime }={\text{Con}}_{1\;\times \;1}^{D}(F^{\prime }),\;F_{H^{\prime }}^{\prime }={\text{Con}}_{1\;\times \;1}^{H}(F^{\prime }),$$ (8)

where $W^{\prime }<W$, $H^{\prime }<H$, $D^{\prime }<D$. Then, compute the covariance matrices of the reduced features for each dimension $W^{\prime }$, $H^{\prime }$, and $D^{\prime }$:

$$C_{W^{\prime }}\in {\mathbb{R}}^{W^{\prime }\;\times \;W^{\prime }},\;C_{H^{\prime }}\in {\mathbb{R}}^{H^{\prime }\;\times \;H^{\prime }},\;C_{D^{\prime }}\in {\mathbb{R}}^{D^{\prime }\;\times \;D^{\prime }}.$$ (9)

Three statistical weight vectors $\lambda_{W}$, $\lambda_{H}$, and $\lambda_{D}$ are generated on each result covariance matrix using row-wise convolution, and these weight vectors are multiplied by the middle feather $F^{\prime }$. The scaled feature maps in three dimensions are fused to generate the output feature map $\hat{F}$:

$$\hat{F}={\text{Con}}_{1\;\times \;1}((\lambda_{W}⊗F^{\prime })⊕(\lambda_{D}⊗F^{\prime })⊕(\lambda_{H}⊗F^{\prime }))⊕F$$ (10)

Then, we introduce the dual-branch loss function(According to Fig 1). The first branch sends *Y*_1_ and *Y*_2_ to an expander $h_{\varphi }$, resulting in embeddings *Z*_1_ and *Z*_2_ (the expander consists of three fully connected layers). Positive samples are different augmentations of the same image, and negative samples are different images in the same batch. The loss function is calculated using the following regularization terms:

$$L_{con}(Z_{1},Z_{2})=\lambda s(Z_{1},Z_{2})+\mu [v(Z_{1})+v(Z_{2})]+\nu [c(Z_{1})+c(Z_{2})],$$ (11)

where *λ*, *μ*, and ν are hyperparameters;

$$s(Z_{1},Z_{2})=\frac{1}{n}\sum_{i=1}^{n}\|z_{1i}-z_{2i}{\|}_{2}^{2},$$ (12)

is the invariance criterion, *n* is the number of images in the batch ;

$$c(Z)=\frac{1}{d}\sum_{i\neq j}[C(Z){]}_{i,j}^{2}$$ (13)

is the covariance regularization term, where *C*(*Z*) is the covariance matrix of *Z*;

$$v(Z)=\frac{1}{d}\sum_{j=1}^{d}\max(0,\gamma -S(z_{j},\epsilon ))$$ (14)

is the variance regularization term, where *d* is the dimensionality of the embedding, *S* is the regularized standard deviation, defined as $S(x,\epsilon )=\sqrt{\text{Var}(x)+\epsilon }$ , and ϵ is a small scalar to prevent numerical instability.

The second branch adopts the idea of online clustering. Features *Y*_1_ and *Y*_2_ are assigned to prototype vectors *C* to obtain “codes" *Q*_1_ and *Q*_2_. The clustering loss is calculated by “exchanging" the prediction problem:

$$L_{clu}(Y_{1},Y_{2})=\ell (Y_{1},Q_{2})+\ell (Y_{2},Q_{1}),$$ (15)

where ℓ(Y,Q) is defined as:

$$\ell (Y,Q)=-\sum_{k}Q(k)\log p(k),$$ (16)

with *p*(*k*) indicating the matching probability of prototype *c*_*k*_ and feature *Y*.

The optimization problem maximizes the similarity between features and prototypes while maintaining smooth coding:

$$\max_{Q}\text{Tr}(Q^{T}C^{T}Y)+\epsilon H(Q),$$ (17)

where *H*(*Q*) is the entropy function of coding *Q*, and *ε* controls the smoothness of the mapping.

## Experiments

In this section, we first compare our method with several CNN-based SSL methods proposed recent on GBCU datasets. Next, we perform an ablation study of the proposed model. Finally, we evaluate the impact of the AL module. All our experiments are tested under the Pytorch framework and run on a computer equipped with a GTX-3060 GPU and an i7-12700@2.10GHz CPU.

**Dataset.** The GBCU dataset, introduced by Basu et al. [39] (https://gbc-iid.github.io/data/gbcu, E-mail: soumen.basu@cse.iitd.ac.in), is a comprehensive collection of 1,255 ultrasound images. It includes 432 normal, 558 benign, and 265 malignant gallbladder cases, all derived from 218 patients. Among these 218 patients, 71, 100, and 47 belong to the normal, benign, and malignant categories, respectively. This meticulously annotated dataset features both image-level labels and bounding box annotations for malignant regions, which is pivotal for advancing gallbladder cancer (GBC) detection research. We report the cross-validation results from ten iterations on the entire dataset, which were used in key experiments to evaluate model generalization. To ensure generalization to unseen patients, during cross-validation, all images from any particular patient appear exclusively in either the training or validation set.

**Experimental setting.** We use the weights of ResNet50 pre-trained on the ImageNet1k dataset as the initial values for some weights in feature extraction, while other parameters are initialized using a normal distribution. The coefficient *β* is set to 1 in Eq (1), $\lambda_{1}$ and $\lambda_{2}$ are set to 1 in Eq (4), Coefficients *α* is set to 0.1 in Eq (5), and *λ* and *μ* are set to 25, and *γ* is set to 1 in Eq (11), *ε* is set to 0.0001 in Eq (17). We use the Adam optimizer to minimize our total loss and train the entire framework for 800 iterations. The batch size is 64, the initial learning rate is 0.003, the momentum is 0.9, the weight decay is $1\;\times \;10^{-6}$, and the learning rate adjustment follows a cosine schedule.

**Evaluation metric.** During the experimental phase of our research, we meticulously assessed the performance of our proposed model through a comprehensive set of key evaluation metrics, including accuracy, Macro-F1 score, sensitivity , specificity and F1 score for the malignant class. Accuracy serves as the cornerstone of measurement standards, depicting the proportion of correct predictions made by the model across all samples. The Macro-F1 score, a simple average of the F1 scores across categories, is an important metric for assessing multi-class models. It takes into account the precision and recall of all classes, thereby reflecting the model’s overall diagnostic capability. We paid particular attention to the identification of the malignant category, aiming to reduce both missed diagnoses and misdiagnosis at the same time. Consequently, we also monitored the sensitivity, specificity, and F1 score for the malignant class to rigorously evaluate the model’s diagnostic performance for this category. By employing these metrics, we strive to conduct a thorough assessment of the model’s effectiveness, ensuring a consistent and reliable evaluation of our algorithm’s capabilities in classifying GBC.

### Comparison experiment

**Fig 3:**
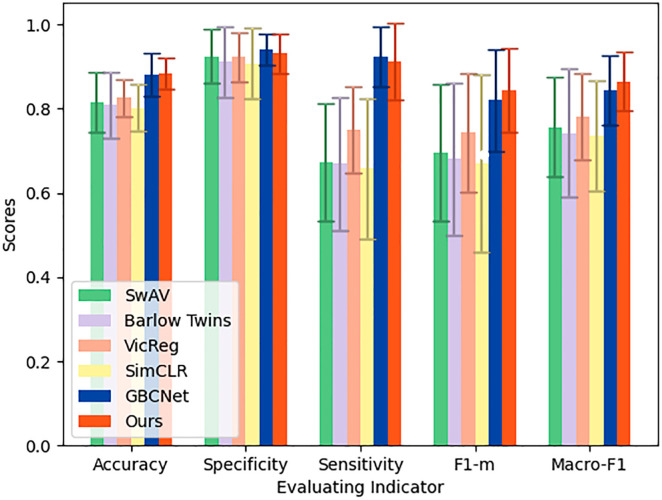
Results of comparison experiment. (Each group represents an evaluation metric, with different colors signifying different algorithms. The height of the bars indicates the magnitude of the metric, and the line segments on top of the bars represent the standard deviation of the metric. The taller the bar, the better the performance; the shorter the line segment, the more stable the model.)

In this section, we compare the performance of our method with several recent algorithms, including SwAV [48], Barlow Twins [49], VicReg [58], SimCLR [54], and the recently introduced GBCNet [34], which is specifically designed for gallbladder cancer detection. The comparison focuses on key diagnostic metrics, including accuracy, specificity, sensitivity, F1-score, and Macro-F1, as summarized in Table 1 and Fig 3.

**Table 1:** Results of comparison experiment. (Sensitivity refers to the proportion of malignant cases that are correctly identified as such, indicating the detection rate of malignant tumors. Specificity refers to the proportion of non-malignant samples that are correctly identified as non-malignant, reflecting the model’s ability to recognize non-malignant samples and prevent misdiagnosis. The F1-score represents the F1 score for malignant classification. Bolded results indicate the best performance. CI_0.95_ represents the 95% confidence interval, $p_{value}$ represents the *p* value of paired t-test with 0.05 significance between ASGBC and other algorithms, and *p*<0.05 indicates that there is significant difference between the two algorithms in this evaluation index.)

Metric		SwAV [48]	Barlow Twins [49]	VicReg [58]	SimCLR [54]	GBCNet [34]	ASGBC(Ours)
Accuracy	mean ± std	0.816 ± 0.070	0.809 ± 0.079	0.826 ± 0.045	0.802 ± 0.056	**0.882 ± 0.051**	0.884 ± 0.038
	CI_0.95_	(0.766, 0.866)	(0.752, 0.866)	(0.794, 0.858)	(0.762, 0.842)	(0.846, 0.918)	(0.857, 0.911)
	$p_{value}$	**0.012**	**0.009**	**0.001**	**0.002**	>0.05	/
Specificity	mean ± std	0.925 ± 0.064	0.911 ± 0.083	0.923 ± 0.059	0.908 ± 0.083	**0.942 ± 0.037**	0.932 ± 0.047
	CI_0.95_	(0.879, 0.971)	(0.852, 0.970)	(0.881, 0.965)	(0.849, 0.967)	(0.916, 0.968)	(0.898, 0.966)
	$p_{value}$	>0.05	>0.05	>0.05	>0.05	>0.05	/
Sensitivity	mean ± std	0.673 ± 0.140	0.669 ± 0.158	0.750 ± 0.102	0.658 ± 0.166	**0.923 ± 0.071**	0.912 ± 0.091
	CI_0.95_	(0.573, 0.773)	(0.556, 0.782)	(0.673, 0.827)	(0.539, 0.777)	(0.872, 0.974)	(0.847, 0.977)
	$p_{value}$	**0.003**	**0.000**	**0.007**	**0.002**	>0.05	/
F1-Score	mean ± std	0.695 ± 0.162	0.681 ± 0.193	0.743 ± 0.141	0.670 ± 0.210	0.820 ± 0.121	**0.844 ± 0.101**
	CI_0.95_	(0.579, 0.811)	(0.543, 0.819)	(0.642, 0.844)	(0.520, 0.820)	(0.733, 0.907)	(0.772, 0.916)
	$p_{value}$	**0.004**	**0.003**	**0.009**	**0.006**	>0.05	/
Macro-F1	mean ± std	0.757 ± 0.119	0.742 ± 0.144	0.782 ± 0.097	0.736 ± 0.132	0.843 ± 0.083	**0.865 ± 0.069**
	CI_0.95_	(0.672, 0.842)	(0.639, 0.845)	(0.713, 0.851)	(0.642, 0.830)	(0.784, 0.902)	(0.816, 0.914)
	$p_{value}$	0.053	**0.031**	**0.040**	**0.047**	>0.05	/

Our method, ASGBC, achieves the highest performance in most metrics. Specifically, it attains an accuracy of 0.884  ±  0.038 (95% CI: 0.857–0.911), outperforming all other methods with statistically significant differences (all *p*<0.05).

In terms of sensitivity, ASGBC reaches 0.912  ±  0.091 (95% CI: 0.847–0.977), significantly higher than SwAV, Barlow Twins, VicReg, and SimCLR (all *p*<0.01), indicating a superior ability to detect malignant cases. Although GBCNet achieves a slightly higher sensitivity 0.923  ±  0.071, the difference is not statistically significant (*p*>0.05). Notably, ASGBC achieves comparable performance using only 35% of the labeled data, highlighting its efficiency and practical value in clinical settings where annotated data are scarce.

In terms of specificity, ASGBC achieves 0.932  ±  0.047 (95% CI: 0.898–0.966), which is among the second highest, though not statistically different from other methods (*p*>0.05). This suggests that our method maintains a strong ability to correctly identify non-malignant cases, reducing the risk of misdiagnosis.

The F1-score and Macro-F1, which reflect the balance between precision and recall, further demonstrate the robustness of our method. ASGBC achieves an F1-score of 0.844  ±  0.101 (95% CI: 0.772–0.916) and a Macro-F1 of 0.865  ±  0.069 (95% CI: 0.816–0.914), both of which are the highest among all compared methods. The improvements over SwAV, Barlow Twins, VicReg, and SimCLR are statistically significant (all *p*<0.05). Again, while GBCNet performs slightly worse in these metrics, the difference is not statistically significant (*p*>0.05).

Moreover, the low standard deviations observed in ASGBC’s metrics (e.g., accuracy SD = 0.038, Macro-F1 SD = 0.069) indicate that our model provides stable and reliable predictions across different test folds. In contrast, methods like Barlow Twins and SimCLR exhibit higher variability, which may limit their clinical applicability.

These results suggest that our method not only achieves superior diagnostic accuracy but also maintains robustness and generalizability. The integration of multi-scale feature extraction and dual-branch loss optimization contributes to its strong performance. Furthermore, the reduced dependency on labeled data makes ASGBC a promising tool for real-world clinical deployment, especially in resource-limited settings.

### Clinical relevance and human-AI collaboration

In addition to comparing our ASGBC model with other computational methods, it is essential to evaluate its performance in the context of real-world clinical applications, particularly in relation to human expert diagnostic accuracy. To this end, we reference the human baseline performance reported by Basu et al. [39], where two experienced radiologists independently evaluated the same dataset used in our experiments. As shown in Table 2, Radiologist A achieved an accuracy of 0.816, specificity of 0.873, and sensitivity of 0.707, while Radiologist B achieved an accuracy of 0.784, specificity of 0.911, and sensitivity of 0.732. In comparison, our ASGBC model significantly outperforms both radiologists across all three metrics, achieving an accuracy of 0.884, specificity of 0.932, and sensitivity of 0.912.

**Table 2:** Performance comparison between ASGBC and human radiologists.

Method	Accuracy	Specificity	Sensitivity
Radiologist A	0.816	0.873	0.707
Radiologist B	0.784	0.911	0.732
ASGBC	0.884	0.932	0.912

To further assess the diagnostic consistency between our model and human experts, we conducted a Kappa consistency analysis. Due to budget constraints, we were unable to organize a new blind test involving multiple radiologists. Instead, we referenced the blind test data from the original dataset publication, which included diagnostic results from two radiologists. Since individual diagnostic results for each image were not available, we employed a mathematical derivation to estimate the Kappa coefficient. The detailed derivation is provided in the Appendix.

As shown in Table 3, the Kappa coefficient ranges from 0.434 to 0.766 for Radiologist A and from 0.502 to 0.837 for Radiologist B. These results indicate that ASGBC demonstrates a stronger alignment with Radiologist B compared to Radiologist A. Importantly, the worst-case Kappa values for both radiologists exceed the minimum clinical threshold of 0.40, suggesting that the consistency is acceptable in both cases. Furthermore, the best-case Kappa values for both radiologists meet the diagnostic independence standards (>0.75), which implies that the consistency between the radiologists and ASGBC is strong and reliable. Overall, these findings highlight the robustness of ASGBC in achieving high consistency with human expert assessments. These results demonstrate that our model not only excels in computational benchmarks but also holds significant potential for enhancing clinical diagnostic accuracy. The high sensitivity of ASGBC (0.912) is particularly noteworthy, as it indicates a strong capability to correctly identify malignant cases, thereby reducing the risk of missed diagnoses—a critical factor in early-stage gallbladder cancer detection.

**Table 3:** Kappa consistency ranges.

Comparison	$\kappa_{\text{min}}$	$\kappa_{\text{max}}$
Radiologist A vs ASGBC	0.434	0.766
Radiologist B vs ASGBC	0.502	0.837

To further bridge the gap between AI performance and clinical utility, we envision the development of a real-time lesion prompting system that integrates the diagnostic strengths of ASGBC with the expertise of human clinicians. In practice, such a system would assist radiologists by providing rapid diagnostic suggestions and highlighting suspicious regions during ultrasound examinations. This collaborative approach would not only improve diagnostic speed and accuracy, especially in complex or early-stage cases, but also reduce the workload and cognitive burden on medical professionals.

Importantly, this human-AI partnership does not seek to replace clinicians but rather to augment their capabilities. By combining the computational efficiency and consistency of AI with the contextual judgment and experience of human experts, we aim to create a synergistic diagnostic workflow that maximizes the strengths of both. Ultimately, we believe that ASGBC can serve as a powerful clinical assistant, contributing to more accurate, efficient, and accessible medical diagnostics, and ultimately improving patient outcomes.

### Noise robustness analysis

To evaluate the robustness of the proposed ASGBC model under noise interference, we conducted experiments by adding Gaussian noise with a kernel sigma of 5 to the B-ultrasound images. The performance metrics under different noise levels are presented in Table 4.

**Table 4:** Performance metrics under different noise levels.

Noise level	Accuracy	↓	Specificity	↓	Sensitivity	↓	F1-score	↓	Macro-F1	↓
0%	0.884	-	0.932	-	0.912	-	0.844	-	0.865	-
1%	0.875	1.02%	0.923	0.97%	0.903	0.99%	0.836	0.95%	0.856	1.04%
5%	0.862	2.49%	0.909	2.47%	0.888	2.63%	0.823	2.49%	0.843	2.54%
10%	0.859	2.83%	0.905	2.90%	0.885	2.96%	0.820	2.84%	0.840	2.89%

The results demonstrate that the ASGBC model maintains relatively stable performance under low noise levels. Specifically, with 1% Gaussian noise, the accuracy decreases by only 1.02%, specificity by 0.97%, sensitivity by 0.99%, F1-score by 0.95%, and Macro-F1 by 1.04%. As the noise level increases to 5%, the performance degradation becomes more pronounced, with accuracy decreasing by 2.49%, specificity by 2.47%, sensitivity by 2.63%, F1-score by 2.49%, and Macro-F1 by 2.54%. At 10% noise level, the model still retains reasonable performance, with accuracy at 0.859, specificity at 0.905, sensitivity at 0.885, F1-score at 0.820, and Macro-F1 at 0.840.

These findings indicate that the ASGBC model exhibits good robustness against Gaussian noise, which is crucial for clinical applications where ultrasound images may be affected by various noise artifacts. The model’s ability to maintain performance under noise interference highlights its potential for real-world deployment in clinical settings.

### Ablation study

**Fig 4:**
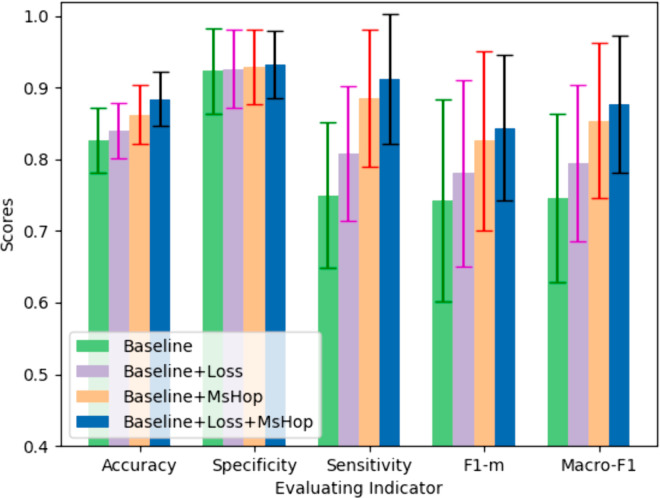
Results of ablation study.

We use the VicReg [58] model with the best performance from the comparative experiment as the baseline. Its feature extractor is the backbone of ResNet, and the loss function only includes *L*_*con*_. The results presented in the Table 5 and Fig 4 provide insights into the impact of the proposed enhancements on the baseline model for GBC classification using B-ultrasound images.

**Table 5:** Results of ablation study.

Method	Accuracy	Specificity	Sensitivity	F1-score	Macro-F1
Baseline	0.826 ± 0.045	0.923 ± 0.059	0.750 ± 0.102	0.743 ± 0.141	0.746 ± 0.118
Baseline+Loss	0.840 ± 0.039	0.926 ± 0.052	0.808 ± 0.094	0.781 ± 0.125	0.794 ± 0.109
Baseline+MsHop	0.862 ± 0.041	0.928 ± 0.055	0.885 ± 0.096	0.826 ± 0.130	0.854 ± 0.108
Baseline+Loss+MsHop	**0.884 ± 0.038**	**0.932 ± 0.047**	**0.912 ± 0.091**	**0.844 ± 0.101**	**0.877 ± 0.096**

**Baseline:** The baseline model, serving as a reference, achieved an accuracy of 0.826 with a standard deviation of 0.045, a specificity of 0.923 with a standard deviation of 0.059, a sensitivity of 0.750 with a standard deviation of 0.102, a F1 score of 0.743 with a standard deviation of 0.141 and a macro-F1 score of 0.746 with a standard deviation of 0.118. These figures establish a benchmark for the evaluation of the subsequent modifications.

**Independent contribution of dual-branch loss:** The integration of the proposed dual-branch loss function (Loss) into the baseline model has led to a comprehensive enhancement in model performance, with accuracy increasing by 1.7% (from 0.826 to 0.840) and the standard deviation reduced by 13.3% (from 0.045 to 0.039). There is a slight improvement in specificity, reaching 0.926 with a standard deviation of 0.055. Sensitivity is enhanced by 7.7% (from 0.750 to 0.808) with a standard deviation reduced by 7.8% (from 0.102 to 0.094). The F1 score and macro-F1 score have also seen improvements of 5.1% (from 0.743 to 0.781) and 6.4% (from 0.746 to 0.794), respectively. This indicates that the dual-branch loss function contributes to the model’s predictive performance while maintaining its ability to correctly identify negative samples.

**Independent contribution of MsHop:** After introducing the multi-scale high-order pooling module (MsHop) into the baseline model, the five evaluation metrics have been improved to 0.862, 0.928, 0.885, 0.826, and 0.854 respectively, with lower standard deviations compared to the baseline model, showing a more pronounced improvement than the loss function. Specifically, accuracy increased by 4.4% (from 0.826 to 0.862), specificity by 0.5% (from 0.923 to 0.928), sensitivity by 18.0% (from 0.750 to 0.885), F1 score by 11.2% (from 0.743 to 0.826), and macro-F1 score by 14.5% (from 0.746 to 0.854). This suggests that the multi-scale high-order pooling module effectively captures features at different scales, which is important for classification tasks.

**Combined effect:** When both the dual-branch loss function and the multi-scale high-order pooling module are combined with the baseline model, the five evaluation metrics reach 0.884, 0.932, 0.912, 0.844, and 0.877, representing an increase of 7.0%, 1.0%, 21.6%, 13.6%, and 17.6% respectively compared to the baseline model. The standard deviations have decreased by 15.6%, 20.3%, 10.8%, 28.4%, and 18.6%. It can be further observed that the loss module contributes more to the reduction of variance, effectively enhancing the model’s stability, while the multi-scale high-order pooling module contributes more to performance improvement. The combination of these techniques demonstrates a synergistic effect, enhancing the overall classification performance of the GBC model using B-ultrasound images.

### Effect of active learning

**Fig 5:**
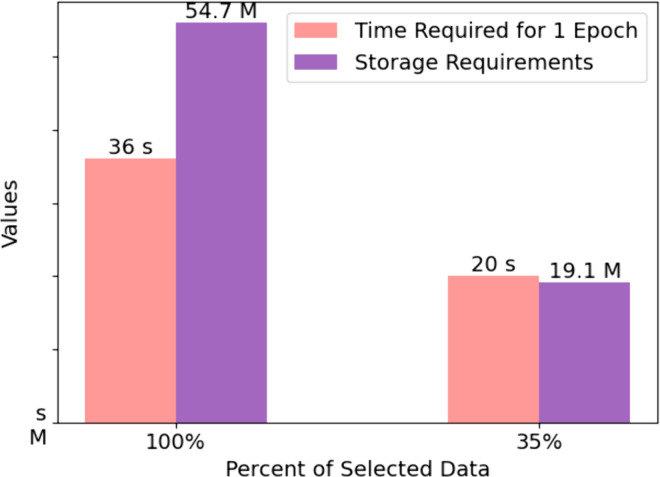
Effect of active learning.

The experimental results presented in Fig 5 offer insights into the benefits of using a subset of data for training deep learning models. With only 35% of the total data, the time required for one training epoch is significantly reduced to 20 seconds, compared to 36 seconds when using the full dataset. This reduction in training time is crucial for rapid prototyping and iterative model refinement. Additionally, the storage requirements are substantially lower, with only 19.1 megabytes needed for 35% of the data, compared to 54.7 megabytes for the complete set. This decrease in storage demand reduces pressure on memory and storage, facilitates more efficient data management, and can lower costs associated with data storage and processing. These advantages underscore the potential for a more sustainable and cost-effective approach to deep learning model development, especially in scenarios where computational resources are limited or rapid model deployment is desired.

**Fig 6:**
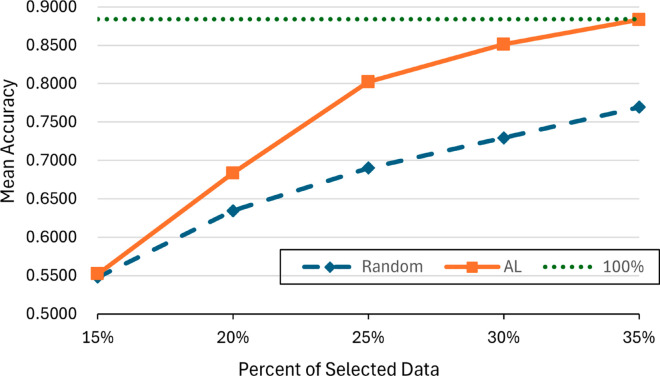
Comparison between AL and randomly selected data.

The experimental analysis shown in Fig 6 reveals a stark contrast between random data selection and AL strategies. Initially, at 15% data selection, algorithm with AL achieves a modest accuracy of 0.5479, comparable to random selection. However, as the percentage of data increases, AL demonstrates a superior ability to enhance model accuracy, reaching an impressive 0.8023 accuracy with just 25% of the data. This trend continues, with accuracy peaking at 0.8831 when 35% of the data is utilized, significantly outperforming random selection, which stabilizes at 0.8839 even with the full dataset. These results underscore the efficacy of AL in optimizing model performance with a fraction of the data, highlighting its potential for efficient and targeted data acquisition in machine learning processes.

The dataset used in this study was collected from PGIMER, a tertiary care referral hospital located in Chandigarh, northern India. All ultrasound images were acquired by radiologists using the Logiq S8 system. While this dataset provides a valuable foundation for model development, we acknowledge that its single-center origin may limit the generalizability of the model. Variations in ultrasound equipment brands and models across different hospitals could potentially affect model performance when applied in new clinical settings. For instance, differences in image resolution, contrast, and noise levels among devices may lead to performance degradation in environments that differ from the training data.

In addition, patient demographics and disease distributions may vary across institutions, including factors such as age, gender composition, and prevalence of specific conditions. These discrepancies could further influence the model’s generalization ability.

To address these limitations, we have outlined a comprehensive plan for future external validation. With sufficient funding, we aim to collaborate with multiple medical centers to conduct multi-institutional validation studies. This will allow us to evaluate the model’s performance across a broader range of ultrasound devices, including those manufactured by Siemens, Philips, and other vendors. By testing the model in diverse clinical environments, we can better assess its stability and adaptability across different equipment and patient populations.

Furthermore, future work will focus on refining and optimizing the model to enhance its robustness and adaptability under varying conditions. We believe that through multi-center external validation, we can more thoroughly evaluate the clinical utility of the model and provide a more solid foundation for its real-world application.

## Conclusions

This study introduces an innovative approach ASGBC to GBC classification by integrating AL with SSL. The proposed method effectively leverages the advantages of both strategies, reducing the need for annotation and training resources. It achieves accuracy comparable to using the full dataset with just 35% of the data, reducing training time by 44% and memory requirements by 65%. This is particularly valuable in scenarios with limited computational resources or the need for rapid model deployment. The feature extraction module, tailored for the characteristics of B-ultrasound imaging, integrates multi-scale and high-order information retrieval capabilities. The dual-branch loss function considers both data relevance and clustering features, enhancing accuracy and model stability. The classification accuracy, specificity, sensitivity, F1 score, and macro-F1 score achieved 0.884, 0.932, 0.912, 0.844, and 0.877, respectively. Compared to the baseline model, these metrics have seen improvements of 7.1%, 1.0%, 21.5%, 13.6%, and 17.4%, respectively. Additionally, the standard deviations have been reduced by 14.8%, 21.3%, 10.8%, 28.2%, and 19.1%, indicating greater model stability. These enhancements suggest that the proposed algorithm is suitable for clinical applications where precise and timely diagnosis is crucial. Overall, our research contributes to the advancement of medical imaging analysis by providing a practical solution for GBC detection. Future work will continue to refine the model, exploring additional enhancements and broader applications in medical diagnostics. We also plan to explore lightweight variants of the MsHop module, such as channel pruning, efficient convolution designs, or dynamic routing mechanisms, to reduce computational overhead while preserving performance. We will also consider optimizing the overall architecture to balance accuracy and efficiency more effectively.

## Supporting information

S1 Appendix Comprehensive derivation of confusion matrices and Kappa coefficients. (PDF)
