# Supplementary material for: Enhanced gallbladder cancer detection via active and self-supervised learning integration: Innovating B-ultrasound image analysis
Source: PLoS One. 2025 Sep 16;20(9):e0330781. doi: 10.1371/journal.pone.0330781 (PMC12440165; doi:10.1371/journal.pone.0330781)
Supplement: S1 Appendix — (PDF) [file pone.0330781.s001.pdf]

Appendix: Comprehensive Derivation of Confusion Matrices and Kappa Coefficients

The data from the blinded tests of the two radiologists originated from the reference literature. Since we do not have the judgment results of both radiologists for each image, it is impossible to directly calculate the Kappa consistency coefficient. Therefore, We analyze it using mathematical derivation. We calculated the minimum and maximum possible values of the Kappa coefficient under the current experimental setup and results. In fact, the probability of the extreme values occurring is extremely low. The true Kappa coefficient should lie between the theoretical minimum and maximum values.

In this appendix, we provide a detailed methodology for deriving confusion matrices from performance metrics and calculating the minimum and maximum Cohen's Kappa coefficients (κ) for two radiologists (A and B) and the ASGBC algorithm. The calculations are based on the following assumptions and conditions:

1. Binary classification: Malignant (class 2) versus Non-malignant (classes 0 + 1).

2. Test set distribution: 122 samples, comprising 42 malignant and 80 non-malignant cases.

3. Radiologists' results are reported as integers, while ASGBC's results are presented as decimals (averaged from 10-fold cross-validation).

Table 6 summarizes the performance metrics for the radiologists and the ASGBC algorithm, which serve as the basis for the subsequent derivations.

Table 6. Performance metrics for radiologists and ASGBC

Method	Accuracy	Specificity	Sensitivity
Radiologist A	0.816	0.873	0.707
Radiologist B	0.784	0.911	0.732
ASGBC	0.884	0.932	0.912

A.1 Definitions and Notation

In order to make the following discussion more coherent, we first define the meanings of the symbols used in the appendix.

- D: a radiologist (either A or B);
- alg: the ASGBC algorithm;
- $N_m = 42$: the number of malignant samples in the test dataset;
- $N_n = 80$: the number of non-malignant samples in the test dataset;
- $N = 122$: the total number of samples in the test dataset;
- TP_D: True positives for radiologist D;
- FP_D: False positives for radiologist D;
- FN_D: False negatives for radiologist D;
- TN_D: True negatives for radiologist D;
- TP_{alg}: True positives for ASGBC;
- FP_{alg}: False positives for ASGBC;
- FN_{alg}: False negatives for ASGBC;
- TN_{alg}: True negatives for ASGBC;
- P_o: the actually observed consistent proportion;
- P_e: the expected consistent proportion under random conditions;
- min_con_m: the minimum consistency of malignant group;
- max_con_m: the maximum consistency of malignant group;
- min_con_n: the minimum consistency of non-malignant group;

- $\max\_con_n$: the maximum consistency of non-malignant group.

A.2: Derivation of Confusion Matrices

A.2.1. Fundamental Equations

To derive the confusion matrix elements for each method, we utilize the following fundamental equations:

$$\begin{aligned}\text{Sensitivity} = & \frac{TP}{TP+FN} = \frac{TP}{42}, \\ \text{Specificity} = & \frac{TN}{TN+FP} = \frac{TN}{80}, \\ \text{Accuracy} = & \frac{TP+TN}{122}.\end{aligned} \tag{18}$$

These equations allow us to calculate the true positives (TP), false positives (FP), false negatives (FN), and true negatives (TN) based on the given performance metrics.

A.2.2. Confusion Matrixes

For Radiologist A, we apply the fundamental equations to derive the confusion matrix elements:

$$\begin{aligned}TP_A = & \text{ Sensitivity} \times 42 = 0.707 \times 42 = 29.694 \approx 30, \\ TN_A = & \text{ Specificity} \times 80 = 0.873 \times 80 = 69.84 \approx 70, \\ FN_A = & \text{ } 42 - TP_A = 42 - 30 = 12, \\ FP_A = & \text{ } 80 - TN_A = 80 - 70 = 10.\end{aligned} \tag{19}$$

For Radiologist B, the confusion matrix elements are derived as follows:

$$\begin{aligned}TP_B = & \text{ Sensitivity} \times 42 = 0.732 \times 42 = 30.744 \approx 31, \\ TN_B = & \text{ Specificity} \times 80 = 0.911 \times 80 = 72.88 \approx 73, \\ FN_B = & \text{ } 42 - TP_B = 42 - 31 = 11, \\ FP_B = & \text{ } 80 - TN_B = 80 - 73 = 7.\end{aligned} \tag{20}$$

For the ASGBC algorithm, the confusion matrix elements are calculated without rounding, as the results are presented as decimals:

$$\begin{aligned}TP_{alg} = & \text{ } 0.912 \times 42 = 38.304, \\ TN_{alg} = & \text{ } 0.932 \times 80 = 74.56, \\ FN_{alg} = & \text{ } 42 - 38.304 = 3.696, \\ FP_{alg} = & \text{ } 80 - 74.56 = 5.44.\end{aligned} \tag{21}$$

Table 7 summarizes the derived confusion matrices for Radiologist A, Radiologist B, and the ASGBC algorithm.

Table 7. Derived confusion matrices

Method	TP	FP	FN	TN
Radiologist A	30	10	12	70
Radiologist B	31	7	11	73
ASGBC	38.304	5.44	3.696	74.56

A.3: Kappa Coefficient Extremum Calculations

A.3.1. Theoretical Framework

The Cohen's Kappa coefficient [2] is defined as:

$$\kappa = \frac{P_o - P_e}{1 - P_e}, \tag{22}$$

where P_e is calculated as:

$$P_e = p_{D+} \cdot p_{alg+} + p_{D-} \cdot p_{alg-}. \tag{23}$$

Here, the marginal probabilities are given by:

$$\begin{aligned} p_{D+} &= \frac{TP_D + FP_D}{N}, \quad p_{D-} = \frac{FN_D + TN_D}{N}, \\ p_{alg+} &= \frac{TP_{alg} + FP_{alg}}{N}, \quad p_{alg-} = \frac{FN_{alg} + TN_{alg}}{N}. \end{aligned} \tag{24}$$

Given that P_e is a constant, the minimum and maximum values of the Kappa coefficient can be expressed as:

$$\begin{aligned} \kappa_{\min} &= \frac{P_o^{\min} - P_e}{1 - P_e}, \\ \kappa_{\max} &= \frac{P_o^{\max} - P_e}{1 - P_e}. \end{aligned} \tag{25}$$

To determine these bounds, we need to consider the minimum and maximum possible values of the actually observed consistent proportion P_o. Specifically, we have:

$$\begin{aligned} P_o^{\min} &= \frac{\text{min\_con}_m + \text{min\_con}_n}{N}, \\ P_o^{\max} &= \frac{\text{max\_con}_m + \text{max\_con}_n}{N}. \end{aligned} \tag{26}$$

For the malignant group, the minimum consistent value min_con_m and the maximum consistent value max_con_m are given by:

$$\begin{aligned} \text{min\_con}_m &= \max(0, TP_D + TP_{alg} - N_m) + \max(0, FN_D + FN_{alg} - N_m), \\ \text{max\_con}_m &= \min(TP_D, TP_{alg}) + \min(FN_D, FN_{alg}). \end{aligned} \tag{27}$$

Similarly, for the non-malignant group, the minimum and maximum consistent values are:

$$\begin{aligned} \text{min\_con}_n &= \max(0, FP_D + FP_{alg} - N_n) + \max(0, TN_D + TN_{alg} - N_n), \\ \text{max\_con}_n &= \min(FP_D, FP_{alg}) + \min(TN_D, TN_{alg}). \end{aligned} \tag{28}$$

Equations (27)(28) are obtained according to the Fréchet bounds [1] for bivariate distributions with fixed marginals. These expressions allow us to calculate the range of possible Kappa values based on the observed consistent proportion, taking into account the constraints imposed by the marginal distributions.

A.3.2 Calculation for Radiologist A vs ASGBC

Substituting the data of the first and third rows in Table 7 into Equations (23) and (24) , we can get:

$$
\begin{aligned}
p_{A+} = &\quad (30+10)/122 \approx 0.3279, \\
p_{A-} = &\quad (12+70)/122 \approx 0.6721, \\
p_{alg+} = &\quad (38.304+5.44)/122 \approx 0.3586, \\
p_{alg-} = &\quad (3.696+74.56)/122 \approx 0.6414, \\
P_e = &\quad (0.3279 \times 0.3586) + (0.6721 \times 0.6414) \approx 0.1176 + 0.4311 = 0.5487.
\end{aligned} \tag{29}
$$

Substituting the data in Table 7 into Equations (27) and (28) , we can get:

$$
\begin{aligned}
\text{min\_con}_m = &\quad \max(0, 30+38.304-42) + \max(0, 12+3.696-42) \\
= &\quad \max(0, 26.304) + \max(0, -26.304) \\
= &\quad 26.304 + 0 = 26.304, \\
\text{max\_con}_m = &\quad \min(30, 38.304) + \min(12, 3.696) \\
= &\quad 30 + 3.696 = 33.696, \\
\text{min\_con}_n = &\quad \max(0, 10+5.44-80) + \max(0, 70+74.56-80) \\
= &\quad \max(0, -64.56) + \max(0, 64.56) \\
= &\quad 0 + 64.56 = 64.56, \\
\text{max\_con}_n = &\quad \min(10, 5.44) + \min(70, 74.56) \\
= &\quad 5.44 + 70 = 75.44.
\end{aligned} \tag{30}
$$

Substituting the calculation results of Equation (30) into Equation (26), we can obtain:

$$
\begin{aligned}
P_o^{\min} = &\quad (26.304+64.56)/122 \approx 0.7448, \\
P_o^{\max} = &\quad (33.696+75.44)/122 \approx 0.8946.
\end{aligned} \tag{31}
$$

Substituting the calculated $P_o^{\min}, P_o^{\max}$ and P_e into Equation (25), we have:

$$
\begin{aligned}
\kappa_{\min} = &\quad \frac{0.7448-0.5487}{1-0.5487} = \frac{0.1961}{0.4513} \approx 0.434, \\
\kappa_{\max} = &\quad \frac{0.8946-0.5487}{1-0.5487} = \frac{0.3459}{0.4513} \approx 0.766.
\end{aligned} \tag{32}
$$

A.3.3 Calculation for Radiologist B vs ASGBC

Similar to Section A.3.2, substituting the data of the second and third rows in Table 7 into Equations (23) and (24) , we can get:

$$
\begin{aligned}
p_{B+} = &\quad (31+7)/122 \approx 0.3115 \\
p_{B-} = &\quad (11+73)/122 \approx 0.6885 \\
p_{alg+} = &\quad 0.3586 \quad \text{(same as before)} \\
p_{alg-} = &\quad 0.6414 \quad \text{(same as before)} \\
P_e = &\quad (0.3115 \times 0.3586) + (0.6885 \times 0.6414) \approx 0.1117 + 0.4416 = 0.5533
\end{aligned} \tag{33}
$$

Substituting the data in Table 7 into Equations (27) and (28) , we can get:

$$\begin{aligned}
\text{min_con}_m &= \max(0, 31 + 38.304 - 42) + \max(0, 11 + 3.696 - 42) \\
&= \max(0, 27.304) + \max(0, -27.304) \\
&= 27.304 + 0 = 27.304 \\
\text{max_con}_m &= \min(31, 38.304) + \min(11, 3.696) \\
&= 31 + 3.696 = 34.696 \\
\text{min_con}_n &= \max(0, 7 + 5.44 - 80) + \max(0, 73 + 74.56 - 80) \\
&= \max(0, -67.56) + \max(0, 67.56) \\
&= 0 + 67.56 = 67.56 \\
\text{max_con}_n &= \min(7, 5.44) + \min(73, 74.56) \\
&= 5.44 + 73 = 78.44
\end{aligned} \tag{34}$$

Substituting the calculation results of Equation (34) into Equation (26), we can obtain:

$$\begin{aligned}
P_o^{\min} &= (27.304 + 67.56)/122 \approx 0.7776 \\
P_o^{\max} &= (34.696 + 78.44)/122 \approx 0.9274
\end{aligned} \tag{35}$$

Substituting the calculated $P_o^{\min}, P_o^{\max}$ and P_e into Equation (25), we have:

$$\begin{aligned}
\kappa_{\min} &= \frac{0.7776 - 0.5533}{1 - 0.5533} = \frac{0.2243}{0.4467} \approx 0.502 \\
\kappa_{\max} &= \frac{0.9274 - 0.5533}{1 - 0.5533} = \frac{0.3741}{0.4467} \approx 0.837
\end{aligned} \tag{36}$$

A.3.4 Summary of Results

The Kappa consistency ranges between radiologists and ASGBC are summarized in Table 3. As shown, the Kappa coefficient ranges from 0.434 to 0.766 for Radiologist A and from 0.502 to 0.837 for Radiologist B. These results indicate that the ASGBC algorithm demonstrates a stronger alignment with Radiologist B compared to Radiologist A. Importantly, the worst-case Kappa values for both radiologists exceed the minimum clinical threshold of 0.40, suggesting that the consistency is acceptable in both cases. Furthermore, the best-case Kappa values for both radiologists meet the diagnostic independence standards (>0.75), which implies that the consistency between the radiologists and the ASGBC algorithm is strong and reliable. Overall, these findings highlight the robustness of the ASGBC algorithm in achieving high consistency with the radiologists' assessments.

References

1. Rüschendorf L. In: FréChet-Bounds and Their Applications. Dordrecht: Springer Netherlands; 1991. p. 151–187.

2. Cohen J. A Coefficient of Agreement for Nominal Scales. Educational and Psychological Measurement. 1960;20:37 – 46.
